# Sex Differences in Sexual Motivation Among U.S. Residents 57–85 Years of Age

**DOI:** 10.1007/s10508-025-03149-8

**Published:** 2025-05-19

**Authors:** Elissa H. Patterson, Linda J. Waite, Martha K. McClintock

**Affiliations:** 1 Departments of Psychiatry and Neurology, Institute for Healthcare Policy and Innovation University of Michigan, 9D 9808 University Hospital, University of Michigan Medical School, 1500 E. Medical Center Dr., SPC 5118, Ann Arbor, MI 48104 USA; 2 Department of Sociology, University of Chicago, Chicago, IL USA; 3 Department of Psychology, University of Chicago, Chicago, IL USA

**Keywords:** Sex, Aging, Sexual motivation, Sex differences, National Social Life, Health, and Aging Project (NSHAP)

## Abstract

Sexual motivation includes proceptivity (mental or physical pursuit of sexual gratification) and receptivity (openness to having sex initiated by someone else). The roles of these two components are not well understood in older adults past reproductive age. We quantify these components and their associations with sexual activity along with differences in sex, age, partner status, health, reproductive steroids and other demographic variables collected during home interviews for the National Social Life, Health, and Aging Project’s nationally representative sample of 3005, 57–85 years old community-dwelling U.S. residents. The majority of older adults had sex and age had only a modest association with their sexual motivation. Proceptive and receptive sexual motivation were independent of each other and each was associated with higher odds of having sex more frequently. Relative to women, men reported higher levels of sexual proceptivity, controlling for demographic and biological variables such as medications, diseases, education, partner status, reproductive steroid levels, and age. Women reported higher sexual receptivity than did men. Although men had higher free salivary testosterone levels, it was associated with higher proceptivity and receptivity within both men and women. DHEA and estradiol were also associated with variations in sexual motivation. Nonetheless, sex differences in each component of sexual motivation remained after controlling for reproductive steroid levels along with demographics and partner status. Having a positive emotional relationship was associated with higher proceptive sexual motivation in women but not in men.

## Introduction

Although sexual activity of both sexes declines with age, many older adults continue to be sexually active during the post-reproductive phase of their lives (Gray & Garcia, 2012; Laumann et al., 2005). Multiple large-scale studies describe the variation in human sexual health and dysfunction of older adults both in the US (National Social Life Health and Aging Project (NSHAP): Laumann & Waite, 2008) and across the globe (Global Study of Sexual Attitudes and Behaviors (GSSAB): Laumann et al., 2005). While physical factors contribute to sexual dysfunction (Lewis et al., 2004, 2010), social context is also an important factor in the frequency of sexual behavior (Freak-Poli, 2020; Gray & Garcia, 2012; Karraker et al., 2011).

Less is known about people’s sexual motivation, as opposed to the incidence of various sexual behaviors, particularly in older adults. Empirically tested theories of human sexual motivation and norms would enrich our understanding of the breadth and variation in human sexuality. In mammals, it is well established that there are multiple components of sexual motivation, with different underlying neuroendocrine circuits, but less is known about these distinct motivational systems in humans. This extensive behavioral and neuroendocrine animal literature has identified two qualitatively different components of sexual motivation that serve as a theoretical model for contributors to human variation in sexual motivation. Proceptivity is pursuing potential mates, approaching and soliciting them to initiate mating and sexual gratification. Receptivity is the degree of responsiveness to courting signals or sexual behavior from potential mates. Both males and females exhibit each type of sexual motivation (Galinsky et al., 2014), although the term “proceptive” was originally proposed only for females (Beach, 1976) and receptivity in males has been relatively understudied.

Norway rats, among many species, exemplify these components of sexual motivation, and are used here to illustrate the distinctions in both behavior and neuroendocrine mechanisms, which is a conceptual framework for understanding human sexuality. A proceptive male approaches a female and chases her while maintaining a steady distance and emitting ultrasound calls until he touches her flanks and mates. Females also actively solicit a preferred male partner. After coming into heat (termed estrus), she approaches the male, grooming him, and then hops and darts away, enticing him to follow. She repeats her solicitations until eventually he follows and they mate (Jennings & Lecea, 2020; McClintock & Adler, 1978). Even without being proceptive, female rats may be receptive to mating and not fight off a male entering her space. When he mounts and simply touches her sensitized flanks, she permits mating by adopting a lordosis posture (Pfaff et al., 2008). Likewise, some males are not receptive to female solicitations and do not follow them (Clark et al., 1985) or are more receptive to solicitations from some females than others, in a form of mate choice (McClintock, 1984).

These two components of sexual motivation involve different neuroendocrine mechanisms, explicated in detail in rats. Proceptivity relies on the preoptic area of the hypothalamus (reviewed by Paredes, 2009; Pfaus et al., 2004; Ventura-Aquino, 2020, 2023), whereas receptivity depends on an intact ventromedial nucleus of the hypothalamus and the central gray area (reviewed by Pfaff et al., 2008). Neuropharmacological treatments can alter one component without altering the other (Guarraci, 2019; Pfaus et al., 2004), indicating their independence.

These two specific components of sexual motivation are also observed across species, even though the form of the behaviors are species specific. In non-human primates, females exhibit both proceptivity and receptivity, which have different hormonal bases (Baum et al., 1977; Dixson, 2002; Jennings & de Lecea, 2020; Rooker, 2020). Male non-human primates also exhibit both components, although the neuroendocrine differences have not been as well studied (Dixson, 2012; Fitzpatrick et al., 2014; Hrdy, 1986; Small, 1993; Wallen, 1995). In humans, each component of sexual motivation is not only manifest by different behaviors, but also experienced as thoughts, desires, and fantasies (Clark & Hatfield, 1989; Mishra, 2023; Pfaus et al., 2003). However, lack of conceptual clarity in defining components of sexual motivation in humans has slowed identifying its brain and hormonal mechanisms (Bittoni & Kiesner, 2023).

Human sexual behavior varies widely, particularly between men and women. Women are reportedly less likely than men to be sexually active at all ages (Galinsky et al., 2014; Laumann et al., 1994; Lindau et al., 2007; Waite et al., 2009). This sex difference raises several questions about sexual motivation. Are there sex differences in the quality or intensity of sexual motivation? Are there sex differences in other variables known to affect sexual activity, such as medications or the functional, neurophysiological, and emotional aspects of disease? Are there sex differences in the effect of being married or living with a partner? Do other demographic factors play a role?

Sex differences in sexual motivation among older adults could also be mediated in part by the established differences in reproductive steroids, e.g., older U.S. men have higher levels of testosterone and progesterone than women (Kozloski et al., 2014). The role of hormones in human sexual motivation is not straightforward. For men, a minimum amount of testosterone is necessary to maintain libido (Baum & Crespi, 2007; Gruenewald & Matsumoto, 2003; Lunenfeld & Nieschlag, 2007; Morales et al., 2004.) The data regarding the role of testosterone in female libido are contradictory; some studies indicate that post-menopausal testosterone supplementation increases libido and some fail to demonstrate a change (Bolour & Braunstein, 2005; Cappelletti & Wallen, 2016; Davis et al., 2004; Kingsberg, 2007; North American Menopause Society, 2005; Schover, 2007).

Until the early 1980s, most academic research models of sexuality focused on heterosexual behaviors, and the predominant assumption in behavioral endocrinology was that female sexual behavior was tightly yoked to male initiation; the female’s role was to be receptive or not (Parsons et al., 1981; Pfaff, 1973). Subsequently, the proceptive role of females soliciting and pacing mating with males was established and conversely, the receptive role of males enacting mate choice (Erskine, 1989; McClintock & Adler, 1978). Since then, the field has grown immensely, reflecting the seismic cultural shifts in the conception of sexuality as well as gender fluidity (Basson, 2000; Janssen & Bancroft, 2023; Quintana & Pfaus, 2024; Wyverkens et al., 2018; Zucker, 2024). Laumann completed national and international surveys of sexual behavior in people (Laumann et al., 1994; Laumann & Michael, 2001; Parish et al., 2007) that laid the foundation enabling us to utilize a model of sexual motivation that quantifies proceptive roles for both men and women, as well as receptive roles for each.

Both proceptive and receptive sexual motivation were measured in The National Social Life, Health, and Aging Project (NSHAP), the first comprehensive large-scale study examining sexual behavior, motivation, dysfunction, and norms of older US adults living at home (ages 57–85)(Lindau et al., 2007; Waite et al., 2009). The NSHAP survey extends the findings of the GSSAB by measuring not only sexual activity but also two specific components of sexual motivation along with a suite of targeted questions. However, this was not a study of sexual motivation around the time of a specific sexual encounter. Rather, NSHAP measured sexual motivation in general, as remembered during the interview. NSHAP is also the first survey to measure levels of free reproductive steroids from a nationally representative sample of older US adults (Gavrilova & Lindau, 2009).

We first tested whether there is an association between both components of sexual motivation and sexual activity in older adults. Then, we assessed with multiple regression the factors known to affect sexual motivation in younger adults: medications, physical and mental health status, demographic variables, religion, education, marital status, and age. Controlling for these variables, we quantified sex differences in motivation as well as the associations with steroids within each sex and having a positive emotional relationship with their partner. This study provides a description of two components of sexual motivation in older US adults and evaluates their social, biological, and psychological associations, identifying sex differences.

## Method

### Participants

The NSHAP interviewed a nationally representative sample of older adults (57 to 85 years of age; born 1920–1947; n = 3005; Round 1) living at home in the USA. This was the initial data collection of a longitudinal multi-cohort study and set the foundation for future longitudinal analyses of aging within individuals. White women are the most prevalent group among older US adults and could be directly sampled. However, in order to increase the power for analyzing sex, age, and race differences, we oversampled men, the oldest participants (75 to 85 years of age), Blacks, and Hispanics following statistical methods that enabled NSHAP to correct for the oversampling (detailed in O’Muircheartaigh et al., 2009), yielding values representative of all these groups.

### Procedure

In-home interviews were conducted in English and Spanish by professional interviewers between July 2005 and March 2006. The measures reported here were collected via computer assisted personal interview (CAPI) and a salivary biosample (Gavrilova & Lindau, 2009; Kozloski et al., 2014). A leave-behind questionnaire (Smith et al., 2009) included additional questions about sexuality to optimize privacy and response rate.

### Measures

All measures were weighted for the participation rate (75.5%) and the oversampling of small groups. Therefore, the reported results reflect the entire population of older adults living at home in the USA (O’Muircheartaigh et al., 2009). The protocol was approved by the institutional review boards of the University of Chicago and National Opinion Research Center; all participants provided written informed consent.

### Sex and Age

Participants reported their sex (man or woman, the dichotomous categories used by the US Census upon which our sampling was based). Age (years) was used both as a continuous variable or categorized into three age groups: 57–64 (youngest old), 65–74 (middle old), and 75–85 (oldest old) years.

### Frequency of Sexual Activity

Sexual activity was defined as “any mutually voluntary activity with another person that involves sexual contact, whether or not intercourse or orgasm occurs.” Participants were asked whether they had sex within the last year or not (dichotomous variable) and if so, about how often: once a day or more, 3 to 6 times a week, once or twice a week, 2 to 3 times a month, once a month or less, or don’t know (n = 1500); responses were standardized (z-score).

### Components of Sexual Motivation

A detailed interview was conducted about participants’ sexual motivation and practices, including elements of dysfunction. These questions were asked after rapport was established during a comprehensive interview about their social networks. Questions were asked with a CAPI, a private anonymized written questionnaire, and a longer written questionnaire completed after the interview (Lindau et al., 2007). Here, we focus on sexual motivation in the participants with a primary sexual relationship as well as those who were not recently sexually active (see Appendix 1 for wording of each specific question).

#### Proceptive Sexual Motivation

Proceptive sexual motivation, the mental or physical pursuit of sexual gratification, was measured with four conceptually related thoughts and behaviors (measures): high frequency of sexual thoughts, high importance of sex, more masturbation and more interest in sex (Galinsky et al., 2014; McClintock, 2015; Waite et al., 2009). Frequency of sexual thoughts was coded with a five-point scale: several times/day [5], every day [4], a few times/week [3], a few times/month [2], less than once a month [1]. This question was asked of all participants, and n = 2672 responded. The question, “How important a part of your life would you say that sex is?” was coded with a five-point scale: extremely important [5], very important [4], moderately important [3], somewhat important [2], not at all important [1]. This question was asked of all participants, and n = 2760 responded.

Masturbation frequency was quantified with a 9-point scale and coded as a dichotomous variable: having masturbated within the last year [1] or not [0]. To increase participants’ privacy for this question, it was asked along with other particularly sensitive sexuality questions via a computerized questionnaire; 2572 participants responded.

Interest in sex (i.e., high proceptive sexual motivation). People who did not experience a prolonged period without interest in sex were considered to have higher proceptive sexual motivation [1] than those who did [0]. The wording was tailored to the participant’s recent level of sexual activity (see Appendix 1 for questions). Those who had been sexually active within the last 12 months were asked if they had had no interest in sex for a period of several months (n = 1538 responded). Those who had not had sex in the past 3 months (n = 1310) were asked whether it was because they had had no interest in sex.

#### Composite Proceptive Score

To increase robustness and minimize the number of analyses of potential explanatory variables and covariates, a composite score for proceptive sexual motivation was created by standardizing the first two items across the representative sample (z-scores) and averaging them: frequency of sexual thoughts and importance of sex (interitem covariance = 0.61; Cronbach alpha reliability coefficient = 0.76). In addition, separate scales were constructed for men and women, each standardized to the sex specific means, for use in sex-specific analyses of reproductive steroids.

The remaining two measures could not be included because they did not have an unbiased response frequency consistent with our representative sample. Although masturbation question was asked of all participants, they completed the private questionnaire non-randomly, causing race/ethnicity-based anomalies in the response-rate (N = 2995; *F*[2.73, 136.31] = 7.06, *p* < 0.0003), confirming previous reports of race/ethnic differences in response rates to sexual questionnaires (Laumann & Michael, 2001; Laumann et al., 1994, 2005). The measure of interest in sex was not asked in the same way across the representative sample and so could not be included in the composite measure of proceptive sexual motivation.

#### Receptive Sexual Motivation

Receptive sexual motivation is being open to having sex initiated by someone else. Individuals can be highly receptive to sexual activity even though it is primarily initiated by their partners. In this context, high receptivity is indicated by having sex more often than initially preferred or from a sense of duty or obligation (Galinsky et al., 2014). There were two measures of receptivity. Satisfaction with frequency of sex was assessed, referring to current or last previous partner. Participants who reported having sex “about as often as you would like” were coded as 0. Reports of having sex much more often than preferred were coded as +2, high receptivity because they were receptive to having sex even though it was not their initial preference. Having sex much less often was coded as -2 (n = 2647). Having had sex out of obligation/duty was coded from 0 (never) to 4 (always) and asked only of those people who had sex in the past year (n = 1566).

#### Composite Receptive Score

To streamline our hypothesis-testing, a composite score of receptive sexual motivation was created by summing these two items from the representative sample and standardizing them (z-score) mean_men+women_ = -0.06, SE = 0.03, min = -1.59, max = 3.91).

### Potential Explanatory Variables and Covariates

Physical health was quantified both with comorbid disease burden (number of chronic Charlson diseases [modified], range, 0–18) (Charlson et al., 1987; McClintock et al., 2016) and medications known to affect sexual function (SSRIs, beta-adrenergic blockers, and non-cardioselective beta blockers; Butler & Lewis, 2002; Qato et al., 2009). A survey adapted version (11-items) of the Center for Epidemiological Studies Depression Scale (Payne et al., 2014; Radloff, 1977) was used to measure depressive symptomatology, and responses were standardized and averaged. Race/ethnicity was based on the US Census categories. Religious categories were Catholic, Protestant, Jewish, Non-Judeo-Christian, and none (Allsop et al., 2021; Hogan, 1982; Laumann et al., 1994; McFarland et al., 2011). Education was analyzed dichotomously as having attended at least some college or not.

Respondents were defined as partnered if they had a sexual, intimate, or romantic relationship (67.0% of the NSHAP sample). For those few with multiple partners, data about the primary current partner were used. Of the partnered respondents, 89.5% were married, 3.0% were living with a partner, and 7.6% had a romantic, intimate, or sexual partner with whom they did not live. In our representative sample of older US adults born 1921–1947, 2.3% self-identified as homosexual (Hsieh & Liu, 2021; Hsieh et al., 2021). This small share of our birth cohort precluded statistical comparisons of heterosexual and homosexual participants in our analyses, though questions were designed to be relevant for all types of sexuality.

Emotional satisfaction and happiness with a partner were measured and combined to create a positive emotional relationship score. Of the 2772 participants who answered at least one sexual motivation question, 88% also answered two questions about satisfaction with their current or most recent relationship: 1. How emotionally satisfying is/was your relationship? (Extremely, Very, Moderately, Rarely, Slightly, Not at all, Don’t know) and 2. How happy is/was your relationship? (A 7-point scale anchored by “very happy” and “very unhappy”).

These two questions were standardized across all respondents and averaged to construct a composite positive emotional relationship score (Cronbach’s alpha reliability coefficient = 0.72). However, this composite score could not be included in the comprehensive model because it excluded people without a current or recent partner. Therefore, it is reported as a separate analysis.

### Reproductive Steroids

A single saliva sample sufficient for assaying free levels of four reproductive steroid hormones was provided by 1,908 participants who also provided data on the covariates described above. The four steroids were testosterone, dehydroepiandrosterone, estradiol, and progesterone (pg/ml of saliva). DHEA, the most common reproductive steroid, was included because it decreases with age and is arguably associated with sexual function in men and women (Basson et al., 2019; El-Sakka, 2018). DHEA binds at androgen receptors and is also a prohormone for testosterone and estradiol depending on the tissue.

Detailed methods for saliva collection and steroid immunoassays are published (Gavrilova & Lindau, 2009; Kozlowski et al., 2014). In sum, a 2 ml passive-drool saliva sample was collected during the home interview by a trained interviewer (following the Granger et al., 2007 protocol). To minimize effects of ambient temperature on salivary analyte levels (Whembolua et al., 2006), each sample vial was immediately placed on a cold pack during transport from the interview site to a freezer (− 20 °C) and stored until overnight shipping on dry ice to Salimetrics laboratories (State College, PA). All assays were conducted in duplicate using commercially available immunoassay kits without modification of the manufacturer’s recommended protocol. On average, the assay protocols had intra- and inter-assay coefficients of variation less than 10% and 15%, respectively. The steroid levels were validated both internally and externally. To ensure hormone values were within the natural assay range of these older participants, we excluded from this analytic sample participants whose medication records indicated they were taking bioactive sex hormones: androgens, estrogens, or 5-alpha reductase inhibitors (199 of which 39 were men) (Gavrilova & Lindau, 2009). Weighted data procedures adjusted inferences to US older adults without exogenous steroids.

Because race/ethnicity groups provided saliva samples at different rates (Gavrilova & Lindau, 2009; Portacolone et al., 2020), steroid hormone levels could not be included in the comprehensive models of sexual motivation without reducing their sample size and losing power. Therefore, steroid values are reported with separate analyses, using weighted data and adjusting for response bias.

Because of inherent differences in the way that steroid hormones function in men and women, two methods of modeling sex were employed to evaluate the relationship between hormones and each component of sexual motivation. First, to verify sensitivity of our survey-based measures, we assessed the relationships between each of the hormones and proceptive sexual motivation, independently in men and women using composite measures of proceptive sexual motivation standardized (z-scores) separately for men and women. Second, we repeated this analysis for receptive sexual motivation. Finally, sex was added to regression models of proceptive and receptive sexual motivation that included data from both men and women.

### Statistical Analysis

Procedures for survey-weighted data and effect sizes allowing generalization to the community-dwelling population of adults, age 57–85 years, living in the USA are detailed in O’Muircheartaigh et al. (2009). Three types of analyses were used. (1) Sex and age differences in each component of proceptive and receptive sexual motivation were tested with a series of chi-squared tests for weighted data. Given the large survey design including weighting, as well as stratification and clustering, the significance of these associations is reported with a design-based Pearson *F*-statistic. (2) Comprehensive models to test the effects of covariates and potential mediators of sex and age differences were tested with nested multiple linear regressions, (unstandardized coefficients [B]) utilizing composite measures of proceptive and receptive sexual motivation. Logistic regression was used to predict categorical and ordered outcome variables. Regression assumptions were tested via post-estimation analysis. (3) When response contingencies or response bias precluded including variables in the comprehensive models, additional analyses were performed with targeted subsets of the population. Statistical analyses were conducted using Stata/SE 14.2.

## Results

### Proceptive and Receptive Sexual Motivation: Independent Predictors of Frequency of Sex

Over half (55.1%) of US older adults had sex within the span of a year and an even larger percent (at least 75%) reported one or both types of sexual motivation. As hypothesized, the proceptive and receptive sexual motivation of older adults were independent of each other (multiple linear regression B_Proceptive_ = -0.02; *p* = 0.69) and were equally associated with sexual activity of older US adults (OR_ProSexMot_ = 3.27, CI = 2.34–4.57, *p* ≤ 0.0005; OR_Receptive_ = 3.12, CI = 2.11—4.62, *p* ≤ 0.0005; *F*[18, 33] = 12.87).

The frequency of sexual activity among those who had sex ranged from once a month or less (35.8%) to three or more times per week (6.5%). Again, both proceptive and receptive sexual motivation independently predicted this variation in the frequency of having sex, although the odds ratio for proceptive was more than twice that of receptive (OR_ProSexMot_ = 3.70, CI = 2.94–4.65, *p* ≤ 0.0005; OR_Receptive_ = 1.76, CI = 1.53–2.04, *p* ≤ 0.0005). Men were more likely than women to be sexually active (59.8% of men vs. 40.2% of women; *F*[1, 50] = 93.25, *p* < 0.0001), but there was no sex difference in the frequency of sexual behavior among those who were sexually active (*F*[3.73, 186.31] = 1.16, *p* < 0.33).

### Proceptive Sexual Motivation: Sex and Age Differences

#### Components of Proceptive Sexual Motivation

**Fig. 1:**
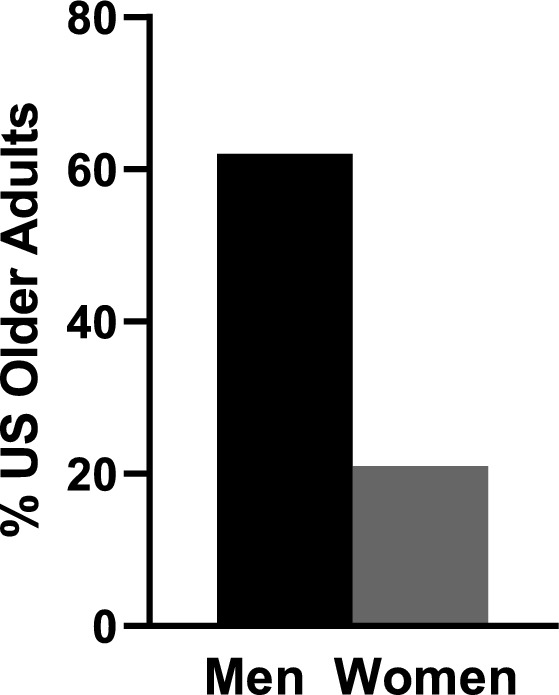
A sex difference in thinking about sex at least once a week among US older adults aged 57–85 years of age

**Fig. 2:**
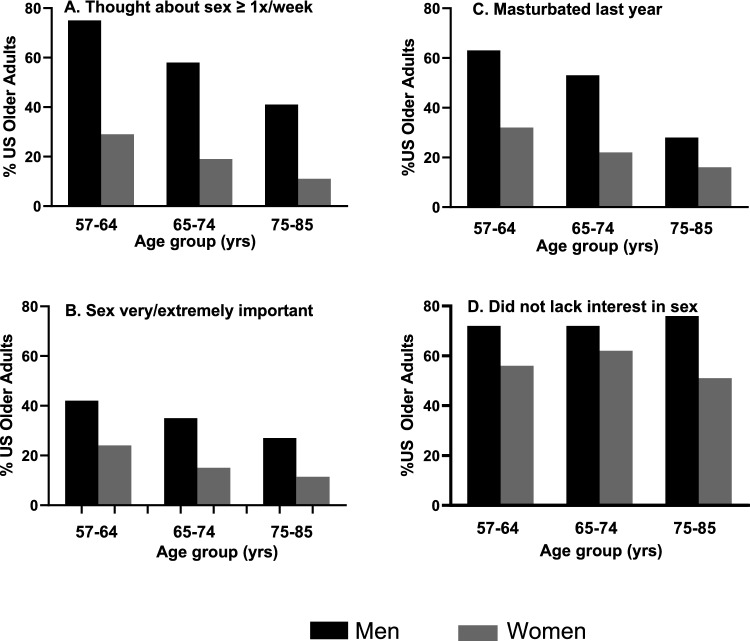
Sex differences in the four components of proactive sexual motivation were manifest in each age group (57–64 years, 65–74 years and 75–85 years). A. Thinking about sex at least once a week. B. Importance of sex C. Masturbating within the past year and D. Interest in having sex. The first three components (A-C) also had significantly lower levels at older ages

Fully 62% of men but only 21% of women reported that they think about sex at least once a week (*F*[1, 63] = 432.27, *p* < 0.00005; see Fig. 1). Specifically, men were more likely than women to report each of the three highest frequency categories (thinking about sex more than once a day, once a day, or once to a few times a week). In contrast, women were more likely to report each of the three lowest frequency categories (thinking about sex once to a few times a month, less than once a month, or never). With increasing age, both men and women thought about sex less frequently (*F* [7.52, 473.61] = 16.34, *p* < 0.0001). Nonetheless, in each age group, men were three times more likely than women to think about sex at least once a week: ages 57–64 (75% vs. 29%, *F*[1, 50] = 217.48 *p* < 0.00005), ages 65–74 (58% vs. 19%, *F*[1, 50] = 93.28, *p* < 0.00005), ages 75–85 (41% vs. 11%, *F*[1, 50] = 64.31, *p* < 0.00005; see Fig. 2A).

The majority of US older adults (75%) indicated that sex is, to some extent, an important part of their lives with only one quarter of the population reporting that sex is not at all important. Twice as many men than women reported that sex is “very important” or “extremely important” (36% vs. 18%) (*F*[1, 50] = 68.03, *p* < 0.00005). From youngest old to oldest old, the sex difference was maintained (Ages 57–64 years: 42% of men vs. 24% of women, *F*[1, 50] = 20.11, *p* < 0.00005); (ages 65–74 years: 35% of men vs. 15% of women, *F*[1, 50] = 31.03, *p* < 0.00005); (ages 75-85 years: 27% of men vs. 12% of women, *F*[1, 50] = 10.02, *p* = 0.0001), even though at older ages both men and women ascribed less importance to sex (*F*[6.68, 420.79] = 15.48, *p* < 0.0001) (see Fig. 2B).

Just over a third of US older adults (38%) reported masturbating within the last year. Men were more likely than women to report that they had (52% vs. 24%, *F*[1, 50] = 149.28, *p* < 0.00005). Although masturbation was lower at older ages (*F*[11.19, 705.1] = 6.99, *p* < 0.0001), even in the 75–85 year age group, 22% of both men and women reported that they had masturbated within the last year (see Fig. 2C).

Of the men and women who had sex within the last year, fewer men than women reported that they went through a several month period during which they lacked interest in sex, indicating low proceptive sexual motivation (28% vs. 43%, *F*[1, 50] = 18.67, *p* = 0.0001, See Fig. 2D). Moreover, if it had been more than three months since they had sex, men were half as likely as women to ascribe it to lacking interest (18% vs. 42%, *F*[1, 50] = 83.11, *p* < 0.0001). There were no age differences in this component: (*F*[1.91, 120.50] = 0.33, *p* = 0.70).

### Proceptive Sexual Motivation: Potential Covariates

Using our composite measure of proceptive sexual motivation, we developed a comprehensive model of covariates that might potentially mediate or confound the observed sex differences in proceptive sexual motivation. We present the results of this hierarchical linear regression in steps below, starting with the simplest model and building up to the final comprehensive model represented in Table 1. Poor physical health (more diagnosed diseases), depression, and using serotonin-selective reuptake inhibitors (SSRIs) were each associated with low proceptive sexual motivation among US older adults, although use of beta-adrenergic blockers, and non-cardioselective beta blockers were not (Model 1, Table 1).

**Table 1:** Proceptive sexual motivation among U.S. residents age 57–85: Covariates and effects of gender, age, and partner status (nested linear regression beta coefficients (SE) n = 2712)

	Model 1	Model 2	Model 3	Model 4	Model 5	Model 6
	Β SE *p*	Β SE *p*	Β SE *p*	Β SE *p*	Β SE *p*	Β SE *p*
Diseases	− 1.06 (0.17) ***	− 1.01 (0.16) ***	− 0.69 (0.14) ***	− 0.42 (0.15) **	− 0.22 (0.15)	− 0.18 (0.147)
Depression	− 0.12 (0.04) **	− 0.12 (0.04) **	− 0.01 (0.03)	− 0.002 (0.03)	− 0.01 (0.03)	− 0.01 (0.032)
SSRI	− 0.19 (0.08) *	− 0.18 (0.08) *	− 0.15 (0.07) *	− 0.19 (0.07) **	− 0.07 (0.06)	− 0.07 (0.064)
Beta Block	0.00 (0.05)	− 0.002(0.05)	0.01 (0.05)	0.03 (0.05)	− 0.01 (0.05)	− 0.02 (0.047)
NCS Beta Bl	0.11 (0.10)	0.12 (0.10)	0.08 (0.09)	0.11 (0.09)	0.07 (0.10)	0.07 (0.093)
*Religion (none)*	referent group	referent group	referent group	referent group	referent group	
Protestant		− 0.17 (0.08) *	− 0.13 (0.08)	− 0.10 (0.07)	− 0.06 (0.07)	− 0.06 (0.07)
Catholic		− 0.19 (0.08) *	− 0.16 (0.07)*	− 0.13 (0.06)	− 0.10 (0.06)	− 0.09 (0.06)
Jewish		0.08 (0.16)	− 0.02 (0.15)	− 0.01 (0.12)	0.10 (0.10)	0.11 (0.10)
Non-Judeo-Christian	− 0.30 (0.12) *	− 0.22 (0.11)*	− 0.21 (0.10)	− 0.13 (0.09)	− 0.12 (0.09)	
*Race (white)*		referent group	referent group	referent group	referent group	referent group
Black		− 0.05 (0.06)	0.07 (0.05)	0.02 (0.05)	0.01 (0.05)	0.02 (0.05)
Hispanic NB		0.01 (0.08)	0.05 (0.07)	0.00 (0.07)	0.02 (0.07)	0.03 (0.07)
Other		− 0.06 (0.13)	− 0.02 (0.09)	− 0.06 (0.10)	− 0.13 (0.11)	− 0.11 (0.11)
Some College			0.26 (0.04) ***	0.22 (0.04) ***	0.18 (0.03) ***	0.18 (0.03) ***
Has Partner			0.71 (0.04) ***	0.64 (0.04) ***	0.46 (0.04)***	1.29 (0.26) ***
Age (1 year)				− 0.02 (0.00) ***	− 0.02 (0.00) ***	− 0.01 (0.00) **
Gender (male)					0.58 (0.03) ***	0.99 (0.43) *
Gender x partner						− 0.25 (0.08) **
Gender x age						− 0.00 (0.01)
Partner x age						− 0.011 (0.004)**
Constant	0.22 (0.03) ***	0.40 (0.08) ***	− 0.36 (0.08) ***	1.10 (0.19) ***	0.88 (0.18) ***	0.17 (0.27)
R-squared ∆	–	0.01	0.15	0.03	0.09	0.01
R-squared	0.03	0.04	0.19	0.22	0.32	0.320

^*^ *p* < 0.05; ^**^ *p* < 0.01; ^***^ *p* < 0.0005

When religious affiliation and race/ethnicity were added to the variables above (Model 2, Table 1), older adults affiliated with Catholic, Protestant, or Non-Judeo-Christian religions each had lower proceptive sexual motivation than those without a religious affiliation, whereas those with a Jewish affiliation were not lower. There were no race/ethnic differences in proceptive sexual motivation.

A separate analysis of masturbation was consistent with higher proceptive sexual motivation among those with a Jewish affiliation or no religious affiliation. Only a third of Catholic, Protestant, and Non-Judeo-Christian older adults reported having masturbated within the last year (35% of Catholics 39% of Protestants, 33% of Non-Judeo-Christians). In contrast, more than half of those with a Jewish affiliation or without any affiliation reported that they had masturbated within the last year (58% and 52% respectively; *F*[3.14, 156.82] = 3.82, *p* = 0.01).

Older adults with more education had higher proceptive sexual motivation (Model 3, Table 1). Because older US men were more likely than women to have attended some college (55.1% of men vs. 46.4% of women, *p* = 0.0003) college attendance could potentially mediate the sex difference in proceptive sexual motivation.

For men and women combined, those with a partner had higher proceptive sexual motivation (Model 3, Table 1). Older US men were more likely than women to have partners (85% of men vs. 61% of women (*F*[1, 50] = 185.67, *p* < 0.0001). Therefore, the apparent sex difference in proceptive sexual motivation could be mediated by differences in having a partner. The effects of a positive emotional relationship with a partner are presented below. Because older age was associated with lower proceptive sexual motivation (Model 4, Table 1) and older adults are more likely to be women (> 75 years are 56% women, *p* < 0.05), age may also mediate the observed sex difference in proceptive sexual motivation.

### Sex Differences and their Potential Mediators

Men had unequivocally higher proceptive sexual motivation than did women when controlling for all of the potential mediating variables mentioned above (proceptive motivation score was 0.58 points higher in men than women; see Model 5 in Table 1; B = 0.576, *F*[16, 35] = 40.62, *p* < 0.0005). Sex accounted for 9.4% of the variability. This was three times the association with older age, which explained only 3% of the variability (the model predicts a 0.021 point decrease in proceptive sexual motivation for each 1 year increase in age (B = -0.021, *F*[15, 36] = 30.30, *p* < 0.0005; R-squared = 0.03). Lastly, in this comprehensive model, having a partner and having attended some college still predicted higher proceptive sexual motivation in men and women, but none of the other covariates remained significant once sex was included in the model (Model 5, Table 1).

### Interactions Among Sex, Partner Status, and Age

**Fig. 3:**
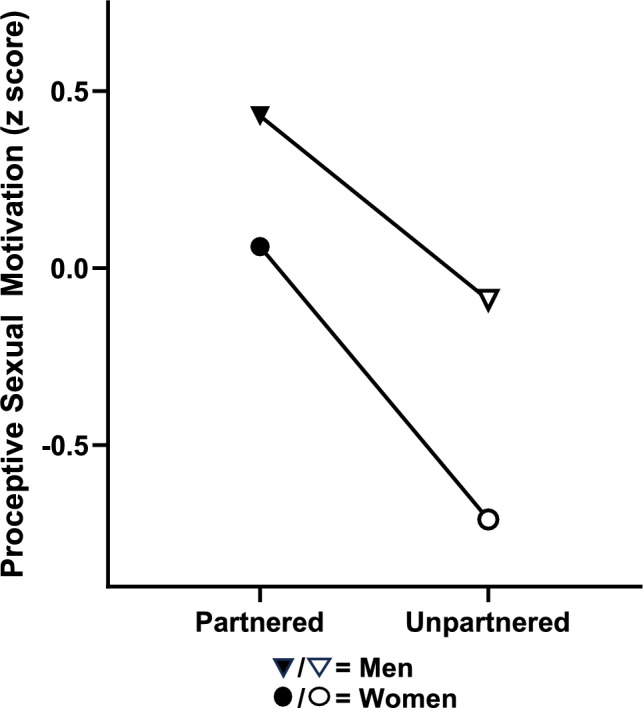
Having a partner was associated with higher proactive sexual motivation in both men and women, an effect that was twice as strong in women than men

Overall, the comprehensive model, which includes interaction terms and all covariates described above, predicted 32% of the variance in proceptive sexual motivation (Model 6, Table 2). Although having a partner was associated with higher proceptive sexual motivation in both men and women, the effect was twice as strong in women (partner status and sex interaction B = -0.253, SE = 0.081, *p* < 0.0001, Model 6, Table 1; women only B_partnerwomen_ = 0.688, SE = 0.054, *p* < 0.0001; men only B_partnermen_ = 0.340, SE = 0.065, *p* < 0.0001; Fig. 3). Moreover, among those with partners, only 15% of men who had not had sex in the past year attributed it to lack of interest compared to 25% of women (*F*[1, 49] = 9.37, *p* = 0.0036). In those without partners, lacking interest in having sex was higher for both men and women, although the sex difference was similar (24% vs. 51%; *F*[1, 49] = 31.80, *p* < 0.0001).

**Table 2:** Receptive sexual motivation among U.S. residents age 57–85: predictor regression coefficients (n = *1417).* Standard errors in parentheses

	Β SE *p*
Gender (male)	− 0.44 (0.09) ***
Age (1 year)	− 0.001 (0.01)
Partner	0.18 (0.08)*
*Health*	
Diseases	− 0.42 (0.33)
Depression	− 0.08 (0.06)
SSRI	0.10 (0.14)
Beta Block	− 0.10 (0.08)
NCS Beta Bl	0.16 (0.12)
*Race/Ethnicity*	
White	referent group
Black	0.52 (0.13)***
Hispanic NB	0.26 (0.14) trend
Non-B, W, HNB	0.07 (0.12)
*Religious Preference*	
No religion	referent group
Protestant	− 0.05 (0.09)
Catholic	0.14 (0.10)
Jewish	− 0.15 (0.15)
Non-Judeo Christian	− 0.09 (0.13)
Educational Level	
Some college	− 0.05 (0.08)

^*^ *p* < 0.05; ^**^ *p* < 0.01; ^***^ *p* < 0.001

**Fig. 4:**
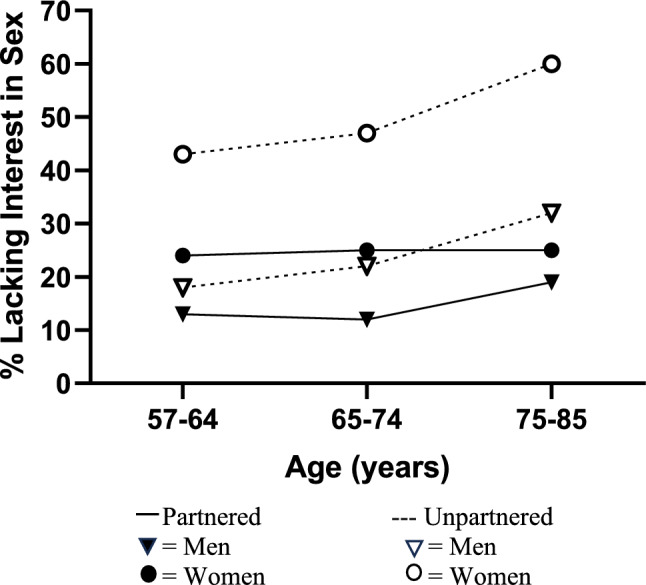
Having a partner was associated with less of the age associated increase in lacking interest in sex, an observation that was stronger in women than men

Having a partner also protected against the lower proceptive sexual motivation seen at older ages (interaction of partner status and age B = -0.011, SE = 0.004, *p* ≤ 0.01 in Model 6, Table 1). Its greatest association was within women, who were insulated from a lack of interest in sex more than men were, especially in the older age groups (*F*[1, 49] = 31.80, *p* < 0.0001, see Fig. 4). Overall, however, sex differences in proceptive sexual motivation were equally strong in all three age groups (interaction of sex and age B = -0.003, SE = 0.006, NS, Model 6, Table 1).

### Receptive Sexual Motivation

People can be highly receptive to having sex even when their proceptive sexual motivation is low. If their partner initiates having sex, they may willingly agree. For example, people may accept a sexual overture to fulfill a role or out of a sense of obligation to their intimate partner. In such instances, they can report that they have had sex more often than they initially preferred.

Although both men and women had sex in the past year out of a sense of obligation or duty, women were more likely to do so (65% of women vs. 43% of men; *F*[3.95, 197.39] = 27.19, *p* < 0.0001). Overall, adults at older ages were less likely to have sex because they felt obliged; and the sex difference disappeared in the oldest age group (men 26% vs. women of 26% women (*F*[3.58, 178.84) = 0.10, *p* = 0.98). Women were almost three times more likely than men to have sex more often than they would prefer (11% vs. 4%; *F*[3.54, 176.83] = 24.19, *p* < 0.0001).

Women were more sexually receptive than men, controlling for the above health, demographic, and social covariates (B_male_ = -0.440, *F*[16, 35] = 7.27, *p* < 0.0001, See Table 2). Sex explained 4.7% of the variance in this comprehensive model of receptive sexual motivation. As with proceptive sexual motivation, having a partner had different effects on the receptivity of men and women (significant interaction of sex and partner status B = -0.373, *F*[19, 32] = 6.80, *p* < 0.01). Among those with partners, men had lower receptive sexual motivation than did women (B_partner_ = -0.485, *F*[15, 36], *p* ≤ 0.0005), whereas without partners, men and women were not significantly different (*p* = 0.235).

### Reproductive Steroid Hormones

#### Proceptive Sexual Motivation and Free Salivary Reproductive Steroids

We first verified that our survey methods could detect the expected associations between free levels of reproductive steroids and proceptive sexual motivation, analyzing individual differences among men and women separately with sex-specific proceptive sexual motivation scales. As expected, men with higher free testosterone had significantly albeit slightly higher proceptive sexual motivation (B = 0.002, *F*[16, 35] = 5.85, 95% CI = 0.0009—0.003, *p* = 0.001). Women had a similar trend (B = 0.002, *F*[16, 35] = 16.07, 95% CI = -0.004—0.0048, *p* = 0.09). Men with higher free DHEA also tended to have higher proceptive sexual motivation (B = 0.001, *F*[16, 35] = 5.92, 95% CI = -0.0001—0.0026, *p* = 0.06), but this was not so in women. Neither free estradiol nor progesterone were associated with proceptive sexual motivation of either men or women (all *p* values ≥ 0.14).

We then tested the hypothesis that the sex differences in free steroid levels (Gavrilova & Lindau, 2009; Kozloski, 2014) mediated the observed sex difference in proceptive sexual motivation. In a series of comprehensive models including both men and women, we added each of the reproductive steroid hormones to the other covariates included in Model 5 Table 1. There was still a sex difference in proceptive sexual motivation even while controlling for free levels of each of the reproductive steroids: (testosterone: B_sex_ = 0.48, *F*[17, 35] = 38.48, *p* < 0.0001; DHEA: B_sex_ = 0.55, *F*[17, 35] = 32.61, *p* < 0.0001; Progesterone: B_sex_ = 0.56, *F*[17, 35] = 43.64,* p* < 0.0001; and Estradiol, B_sex_ = 0.57, *F*[17, 35] = 48.01; *p* < 0.0005 in all four models). These analyses established that absolute levels of free reproductive steroids, detected in a single salivary sample, were not sufficient to explain the observed sex difference in proceptive sexual motivation.

#### Receptive Sexual Motivation and Free Salivary Reproductive Steroids

Free testosterone was positively associated with receptivity in both men and women (*p* = 0.008; B = 0.002). Free DHEA also tended to be higher in women with greater receptivity (*p* = 0.091; B = 0.002), but not in men (*p* = 0.766; B = 0.0003; interaction *p* = 0.059; B = -0.003). The relationship between free estradiol and receptivity differed between men and women (interaction: p = 0.006; B = -0.019). In men, estradiol was positively associated with receptivity but not in women (men: *p* = 0.053; B = 0.008; women: *p* = 0.154; B = -0.007). Free progesterone had no discernable relationship with receptivity.

### Positive Emotional Relationship with Partner

Only among women did feeling emotionally satisfied and happy with their partner predict their proceptive sexual motivation (significant interaction between sex and Positive Emotional Relationship B = -0.126, *F*[18, 33] = 32.70, *p* ≤ 0.001). Women who felt emotionally satisfied and happy about their partner had higher proceptive sexual motivation (B = 0.128, *F*[16, 35] = 17.97, *p* < 0.0005), but in men, their relationship quality had no association (*p* = 0.436).

## Discussion

Sex is an important part of well-being across the lifespan and the majority of older adults are sexually active (Flynn & Gow, 2015; Gray & Garcia, 2012; Lindau et al., 2007; Waite et al., 2009). Sexual behavior, however, is distinct from motivation, which is the internal state of desire or wanting (Baumeister et al., 2012; Berridge, 2018) and is a necessary precursor to consensual sexual behaviors. Here we quantified for the first time two of its independent components, proceptivity and receptivity, in a nationally representative sample of older US adults (aged 57–85, born 1921–1947). In this survey they were measured as remembered during the interview. Identifying the discrete motivations and contingencies at the time of a particular sexual interaction and their interaction with sexual arousal (Basson, 2000; Janssen & Bancroft, 2023) would be an important extension of these concepts, but requires fine-grained temporal data not possible in a survey.

The independence of proceptive and receptive sexual motivation in US older adults is consistent with the extensive mammalian literature demonstrating that they are distinct processes with different neuroendocrine mechanisms (Cummings & Becker, 2012; Hull & Dominguez, 2019; Paredes, 2009; Ventura-Aquino & Agmo, 2023; Ventura-Aquino & Paredes, 2020; Wei et al., 2021). Each component has been quantified in young adults (Clark & Hatfield, 1989; Mishra, 2023; Pfaus et al., 2003). Utilizing this conceptual distinction of motivation in future neuroendocrine research will help distinguish their brain and hormonal mechanisms (Bittoni & Kiesner, 2023; Jennings & de Lecea, 2020).

As expected, including both proceptive and receptive sexual motivation in a regression model predicted sexual activity of older adults. That is, individual differences in either type of sexual motivation translated into differences in both the occurrence and frequency of sexual behavior, controlling for health, reproductive steroids, demographic, and social variables. The direction of causality cannot be determined from a survey approach, but it is plausible that the relationship between sexual motivation and sexual behavior is bidirectional, mediated by sexual arousal (Basson, 2000; Janssen & Bancroft, 2023). The psychological state of proceptive motivation leads to rewarding experiences from sexual behavior, which could drive a positive feedback loop in which the pleasure of sex increases future motivation (Dosch et al., 2016; Goldey et al., 2012). A similar positive feedback loop may increase receptive sexual motivation. Moreover, the multifaceted aspects of sexual activity, including emotional closeness and fulfilling a role along with sexual pleasure would reinforce the likelihood of being receptive in the future.

The questions in NSHAP Round 1 were not optimal for measuring receptive motivation because they didn’t frame its positive aspects. Subsequent NSHAP Rounds incorporated additional questions (e.g., “When your partner wants to have sex with you, how often do you agree?”, Galinsky et al., 2014). Measures of other components of sexual motivation were also subsequently added: sensorimotor function, sexual salience/valence, and sexual attractivity (Galinsky et al., 2014). This richer set of sexual motivation’s components enables future replication and extension of our findings.

The majority (55%) of US older adults in this birth cohort had had sex within the past year, and three-quarters (75%) endorsed sex as an important part of their lives. Despite having similar levels of sexual activity, men and women experienced different types and levels of sexual motivation. Relative to women, men reported greater proceptive sexual motivation, that is, they thought about sex more, they valued it more highly, they self-stimulated more, and they were less likely to have extended periods when they were not interested in sex. Indeed, sex differences accounted for three times the variability accounted for by age (9.3% vs. 3%).

Women reported higher receptive sexual motivation than did men, as reflected in their willingness to have sex more often than they initially preferred and did so out of a sense of obligation to their partners. Having sex out of obligation could represent a desire to sexually satisfy their partner, reinforcing their relationship to fulfill their role as a spouse/partner, or being willing to tolerate sexual activity. All are forms of receptivity. Absence of receptive sexual motivation would be manifest by declining to have sex altogether. Subsequent NSHAP Rounds more precisely target the measures of receptivity enabling further analyses to distinguish among these different forms of sexual receptivity as well as the frequency of sexual abuse among older adults.

Our representative sample of diverse US older adults provided normative data that clinicians, the general public, and geriatricians in particular can use to better understand and validate the sexual experiences of older women and men and the differences between them. Many variables, including hormonal, health-related, social, and demographic, affect sexual behavior (Calabro et al., 2019; Gray & Garcia, 2012; Ricoy-Cano et al., 2020), but may well have different effects on proceptive and receptive sexual motivation. Therefore, they may also help explain the sex differences in sexual motivation. For example, our comprehensive model discussed in detail below explained fully 32% of the variance in proceptive sexual motivation.

Reproductive steroids are known to affect sexual motivation in young adults and animal models (Bullivant et al., 2004; Cappelletti & Wallen, 2016; Pfaff & Saad, 2020; Santi et al., 2018; Ventura-Aquino & Paredes, 2020) raising the hypothesis that the sex differences in the components of sexual motivation could be explained by differences in reproductive steroids. However, although men had higher free testosterone than did women (confirming Kozlowski et al., 2014), testosterone failed to explain the sex differences in proceptive sexual motivation. Likewise, free testosterone levels were not sufficient to explain sex differences in receptive sexual motivation, nor did DHEA, an androgen prohormone. Neither estradiol nor progesterone levels accounted for the sex difference in either component of sexual motivation.

Nonetheless, within each sex, individual variation in reproductive steroids did have significant, albeit small, associations with sexual motivation. Such small associations are also characteristic of sexual motivation in non-human primates (Baum et al., 1978; Phoenix et al., 1973), mammals with extensive sexual experience (Shulman & Spritzer, 2014), and in humans (Cappelletti & Wallen, 2016). Among older men, those with higher free testosterone levels did have significantly, albeit only slightly higher, proceptive sexual motivation, and women showed a similar trend. In addition, women with higher free testosterone, also had greater receptive sexual motivation. This representative survey method enables a large enough sample size to control for important covariates of hormone levels not possible in clinical or laboratory studies. On the other hand, the differences that it does detect may be small and need to be confirmed and interpreted using other research methods. Repeated sampling of hormone levels would provide a more precise estimate of individual differences, possibly revealing a stronger relationship between circulating levels of free reproductive steroids, but that remains for future work. In addition, reproductive steroids could have already created sex differences in types of sexual motivation through their organizational effects on the brain prenatally or earlier in the lifespan as they do in many mammals (Lenz & McCarthy, 2010).

Availability of a partner, whether intimate, romantic, or sexual, is an important determinant of sexual activity (Lindau et al., 2007) and so women’s lower proceptive sexual motivation could simply result from being less likely to have a partner. Indeed, older women were less likely than men to have a partner because women live longer than men in this predominantly heterosexual birth cohort and women tend to be partnered with men older than they are (~ 98% Hsieh & Liu, 2021; Hsieh et al., 2021). However, the sex differences in proceptive sexual motivation were not explained by the presence or absence of a partner (controlling as well for health, hormones, demographic and social variables) even though those with partners did have higher levels of sexual activity and motivation.

The quality of an older woman’s relationship with her partner had a large effect on her sexual motivation. In women, higher emotional satisfaction significantly predicted higher proceptive sexual motivation in marked contrast to men in whom it had no detectable effect. Thus, when considering the effects of partnership status, it is essential to distinguish women that are happy and satisfied with their relationship or not.

In addition to having a partner, higher proceptive sexual motivation was independently associated with being more educated and of a younger age. The age effect was stronger among those without intimate partners. Nonetheless, even in the oldest age group (75–85 years) 41% of men and 11% of women think about sex once a week or more. Moreover, controlling for education, age, and partner status eliminated the differences among religions seen in simpler models of proceptive sexual motivation, and there were no race differences in any model of proceptive sexual motivation. Finally, in a simple model, multimorbidity, depression, and SSRIs were associated with lower proceptive sexual motivation in older adults, but were not, once sex was included in the comprehensive models; this observation indicates that the higher prevalence of these physical and mental health variables among women may drive their apparent overall effects in older adults.

Physical, social, and demographic variables, other than sex, had minimal effects, if any, on receptive sexual motivation. Those with a partner had higher sexual receptivity as did black men and women (for a review of cultural expectations and attitudes, see Laumann & Michael, 2001).

Our findings of sex differences in proceptive and receptive sexual motivation are consistent with the sex differences in the fertility of older men and women (Gray & Garcia, 2012). Men can produce viable gametes well into old age (Martins da Silva & Anderson, 2022). Therefore, in men, proceptive sexual motivation sustained into older ages may serve a direct procreative function. In contrast, women have a finite number of gametes whose viability ends with menopause, around age 50. Nevertheless, older women continue to enjoy both receptive and proceptive sexual motivation beyond menopause. This may serve to strengthen their intimate relationships, stabilize family dynamics, and maintain supportive family relationships increasing the survival of the next generation (Nattrass et al., 2019; Trivers, 1972; Williams, 1957).

These sex differences in sexual motivation are representative of all community-dwelling older adults living in the US and may be considered a normative aspect of aging rather than viewed as an abnormal condition requiring medical treatment. Hypoactive sexual desire syndrome is recognized by the *Diagnostic and Statistical Manual of Mental Disorders* (Fifth ed.), but its definition includes marked distress and interpersonal difficulty. Future work can distinguish typical aging of both proceptive and receptive sexual motivation from pathology and normalize their sex differences. We note that for older adults in the Great Generation it was normative to identify sex dichotomously, and participants were not given the option of non-dichotomous identification. Sampling constraints limited analysis of variation in sexual orientation. Future targeted surveys can look at the intersection of gender identity, sexual orientation, social roles, and other variables that define sex variation in sexual motivation. The analyses reported here provide additional information to fuel the on-going debate about nature vs. nurture with respect to sex differences and behavior in general. Biological drivers are important determinants of behavior, but the well-developed human brain and cortex, in particular, is affected if not shaped by social norms and culture. Such differences are associated with religion, race/ethnicity, time-period in history, cohort, medical advances in reproductive health, and shifting conceptions of sexuality influenced by all of the above. With the foundation built by our analyses of sex differences in two fundamental components of sexual motivation, future research can characterize them more fully, elucidate their relationships to sexual arousal and set them in the social contexts of interpersonal relationships, family, and culture.

## Data Availability

Data from the National Social Life, Health and Aging Project (NSHAP) are publicly available (https://www.icpsr.umich.edu/web/pages/NACDA/nshap.html; https://rcg.bsd.uchicago.edu/nshap/trac/data/browser/wave1/branches/contributors/mb/MBCover) The datasets generated and analyzed in the current study are available from the corresponding author upon reasonable request.

## Appendix 1

NSHAP Wave 1 Sexual Motivation Questions.
